# 7,7′-Dihy­droxy-4,4′-dimethyl-3,4-dihydro-2*H*,2′*H*-4,6′-bichromene-2,2′-dione

**DOI:** 10.1107/S160053681005244X

**Published:** 2010-12-18

**Authors:** P. S. Pereira Silva, Mehtab Parveen, Akhtar Ali, Ali Mohammed Malla, M. Ramos Silva

**Affiliations:** a CEMDRX, Physics Department, University of Coimbra, P-3004-516 Coimbra, Portugal; b Department of Chemistry, Aligarh Muslim University, Aligarh 202002, India

## Abstract

The title compound, C_20_H_16_O_6_, which contains one chiral centre, crystallizes as a racemate. The mean planes of the two coumarin units make a dihedral angle of 88.07 (2)°. The pyrone ring containing the chiral centre adopts a sofa conformation. In the crystal, four mol­ecules are linked by O—H⋯O hydrogen bonds, forming a tetrameric ring with graph-set motif *R*_4_^4^(32). These tetramers are further linked by O—H⋯O hydrogen bonds into a three-dimensional network.

## Related literature

For the chemical reactivity and bioactivity of coumarins and derivatives, see: Fylaktakidou *et al.* (2004 ▶). For a review on bicoumarins, see: Basa (1988 ▶). For the synthesis of bicoumarins, see: Ilyas & Parveen (1996 ▶); Sharma *et al.* (1977 ▶); Gašparová *et al.* (2009 ▶). For the synthesis of the title compound, see: Parveen *et al.* (1991 ▶). For hydrogen-bond motifs, see: Etter *et al.* (1990 ▶).
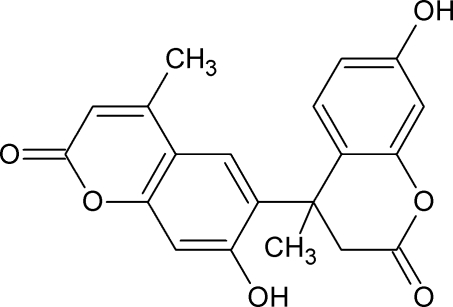


## Experimental

### 

#### Crystal data

C_20_H_16_O_6_
*M* *_r_* = 352.33
Monoclinic, 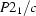

*a* = 9.0432 (2) Å
*b* = 11.5111 (2) Å
*c* = 17.2212 (4) Å
β = 110.870 (1)°
*V* = 1675.06 (6) Å^3^
*Z* = 4
Mo *K*α radiation
μ = 0.10 mm^−1^
*T* = 293 K
0.39 × 0.29 × 0.22 mm

#### Data collection

Bruker APEXII CCD area-detector diffractometer
Absorption correction: multi-scan (*SADABS*; Sheldrick, 2003 ▶) *T*_min_ = 0.863, *T*_max_ = 0.977
44743 measured reflections
4457 independent reflections
3459 reflections with *I* > 2σ(*I*)
*R*_int_ = 0.032

#### Refinement

*R*[*F* ^2^ > 2σ(*F* ^2^)] = 0.040
*wR*(*F* ^2^) = 0.109
*S* = 1.06
4457 reflections
239 parameters
H-atom parameters constrained
Δρ_max_ = 0.24 e Å^−3^
Δρ_min_ = −0.19 e Å^−3^

### 

Data collection: *APEX2* (Bruker, 2003 ▶); cell refinement: *SAINT* (Bruker, 2003 ▶); data reduction: *SAINT*; program(s) used to solve structure: *SHELXS97* (Sheldrick, 2008 ▶); program(s) used to refine structure: *SHELXL97* (Sheldrick, 2008 ▶); molecular graphics: *PLATON* (Spek, 2009 ▶); software used to prepare material for publication: *SHELXL97*.

## Supplementary Material

Crystal structure: contains datablocks global, I. DOI: 10.1107/S160053681005244X/bt5436sup1.cif

Structure factors: contains datablocks I. DOI: 10.1107/S160053681005244X/bt5436Isup2.hkl

Additional supplementary materials:  crystallographic information; 3D view; checkCIF report

## Figures and Tables

**Table 1:** Hydrogen-bond geometry (Å, °)

*D*—H⋯*A*	*D*—H	H⋯*A*	*D*⋯*A*	*D*—H⋯*A*
O3—H3⋯O5^i^	0.82	2.13	2.9406 (13)	169
O6—H6⋯O2^ii^	0.82	1.91	2.7024 (15)	161

Symmetry codes: (i) 
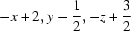
; (ii) 
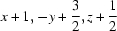
.

**Fig. 1.**
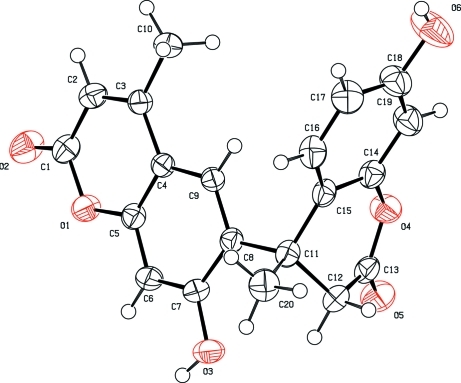
A plot of the title compound. Displacement ellipsoids are drawn at the 50% probability level.

**Fig. 2.**
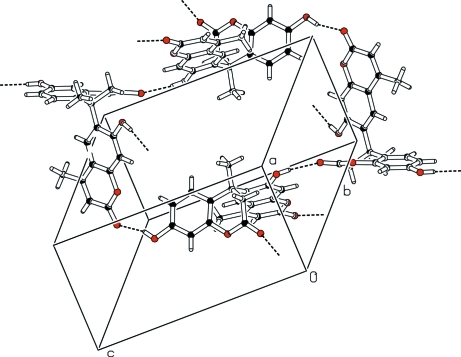
One of the R44(32) rings. The hydrogen bonds are depicted by dashed lines.

## supplementary crystallographic information

### Comment

Studies of natural and synthetic coumarins and its derivatives have been present for a number of years. Coumarins and their derivatives are characterized by excellent chemical reactivity and bioactivity (Fylaktakidou *et al.*, 2004). Bicoumarins are a comparatively new class of naturally occurring compounds (Basa, 1988) and are reputed for their biological activities such as anticoagulant, anticancer, antifungal agents.  Only few bicoumarins have been synthesized (Ilyas & Parveen, 1996; Sharma & Seshadri, 1977; Gašparová *et al.*, 2009). Considering the biological importance and scarcity of work on coumarin dimer a novel coumarin dimer 7,7'-dihydroxy-4,4'-dimethyl-3,4-dihydro-2H,2'H-4,6'- bichromene-2,2'-dione (I) was synthesized by the reinvestigation of synthesis of 7-hydroxy-4-methyl coumarin with the condensation of resorcinol and ethyl acetoacetate in different molar ratio using catalytic amount of polyphospharic acid (PPA) (Parveen *et al.*, 1991). The increase in molar ratio of ethyl acetoacetate leads to a slight increase of coumarin dimer (I).

The title compound, (I), Fig. 1, has one chiral carbon atom (the C11 atom). Both enantiomers are present in the crystal structure, forming a racemate.

In the molecule of (I), the mean planes of the two coumarin units make a dihedral angle of 88.07 (2). In one of the coumarin units, the the dihedral angle between the least-squares planes of the pyrone and benzene rings is 3.36 (6)°. In the other coumarin unit the pyrone ring adopts an envelope conformation and the dihedral angle with the aromatic ring is 13.23 (6)°.

In the crystal, the molecules are linked by O—H···O hydrogen bonds (Fig. 2, Table 2) forming rings with four molecules, graph-set motif *R*_4_^4^(32), according to the Etter's graph-set theory (Etter *et al.*, 1990), centered about inversion centres. These rings are linked, with each molecule participating in two rings, forming a three-dimensional network. The structure is stabilized further by weak C—H···O hydrogen bonds.

### Experimental

Polyphospharic acid was prepared by mixing orthophosphoric acid (15 mL) and phosphorus pentaoxide (23.5 g) followed by heating on a water bath for 1.5 hr. A catalytic amount of polyphosphoric acid (160 g) was added to resorcinol (11 g, 100 mmol) and ethyl acetoacetate (13 mL, 100 mmol) and was heated on a water bath (75–80 °C) for 20 min. with stirring. The viscous mixture was then poured into ice cold water and the resulting solid (18 g, m.p. 180 °C) was crystallized with EtOH as shinning crystal (7 g), it was characterized as 7-hydroxy-4-methyl coumarin by comparison with authentic sample. The mother liquor showed the presence of two bands I&II (TLC, silica-gel, benzene-ethylacetate 2:1) which was separated into individual compounds by preparative thin layer chromatography in the same solvent. The compound I was identified as 7-hydroxy-4-methyl coumarin while the compound II (m.p. 305 °C) was characterized as a novel coumarin dimer, (I), by IR, ^1^H NMR, ^13^C NMR & MS spectra.

### Refinement

All H atoms were located in a difference Fourier synthesis, placed in calculated positions and refined as riding on their parent atoms, using *SHELXL97* (Sheldrick, 2008) defaults.

### Crystal data

**Table:**

C_20_H_16_O_6_	*F*(000) = 736
*M**_r_* = 352.33	*D*_x_ = 1.397 Mg m^−^^3^
Monoclinic, *P*2_1_/*c*	Melting point: 578 K
Hall symbol: -P 2ybc	Mo *K*α radiation, λ = 0.71073 Å
*a* = 9.0432 (2) Å	Cell parameters from 6612 reflections
*b* = 11.5111 (2) Å	θ = 2.5–26.9°
*c* = 17.2212 (4) Å	µ = 0.10 mm^−^^1^
β = 110.870 (1)°	*T* = 293 K
*V* = 1675.06 (6) Å^3^	Block, colourless
*Z* = 4	0.39 × 0.29 × 0.22 mm

### Data collection

**Table:**

Bruker APEXII CCD area-detector diffractometer	4457 independent reflections
Radiation source: fine-focus sealed tube	3459 reflections with *I* > 2σ(*I*)
graphite	*R*_int_ = 0.032
φ and ω scans	θ_max_ = 29.0°, θ_min_ = 2.2°
Absorption correction: multi-scan (*SADABS*; Sheldrick, 2003)	*h* = −12→12
*T*_min_ = 0.863, *T*_max_ = 0.977	*k* = −15→15
44743 measured reflections	*l* = −23→23

### Refinement

**Table:**

Refinement on *F*^2^	Primary atom site location: structure-invariant direct methods
Least-squares matrix: full	Secondary atom site location: difference Fourier map
*R*[*F*^2^ > 2σ(*F*^2^)] = 0.040	Hydrogen site location: inferred from neighbouring sites
*wR*(*F*^2^) = 0.109	H-atom parameters constrained
*S* = 1.06	*w* = 1/[σ^2^(*F*_o_^2^) + (0.0494*P*)^2^ + 0.3328*P*] where *P* = (*F*_o_^2^ + 2*F*_c_^2^)/3
4457 reflections	(Δ/σ)_max_ = 0.001
239 parameters	Δρ_max_ = 0.24 e Å^−^^3^
0 restraints	Δρ_min_ = −0.19 e Å^−^^3^

### Special details

**Table:**

Geometry. All e.s.d.'s (except the e.s.d. in the dihedral angle between two l.s. planes) are estimated using the full covariance matrix. The cell e.s.d.'s are taken into account individually in the estimation of e.s.d.'s in distances, angles and torsion angles; correlations between e.s.d.'s in cell parameters are only used when they are defined by crystal symmetry. An approximate (isotropic) treatment of cell e.s.d.'s is used for estimating e.s.d.'s involving l.s. planes.
Refinement. Refinement of *F*^2^ against ALL reflections. The weighted *R*-factor *wR* and goodness of fit *S* are based on *F*^2^, conventional *R*-factors *R* are based on *F*, with *F* set to zero for negative *F*^2^. The threshold expression of *F*^2^ > σ(*F*^2^) is used only for calculating *R*-factors(gt) *etc*. and is not relevant to the choice of reflections for refinement. *R*-factors based on *F*^2^ are statistically about twice as large as those based on *F*, and *R*- factors based on ALL data will be even larger.

### Fractional atomic coordinates and isotropic or equivalent isotropic displacement parameters (Å^2^)

**Table:**

	*x*	*y*	*z*	*U*_iso_*/*U*_eq_	
O1	0.74647 (10)	0.54064 (8)	0.87909 (5)	0.0368 (2)	
O2	0.54717 (11)	0.46437 (9)	0.90479 (6)	0.0493 (3)	
O3	1.15727 (12)	0.69823 (9)	0.80504 (6)	0.0451 (2)	
H3	1.1142	0.6550	0.7656	0.068*	
O4	1.12946 (11)	1.04007 (8)	0.96142 (5)	0.0417 (2)	
O5	1.02955 (13)	1.03756 (9)	0.82593 (6)	0.0524 (3)	
O6	1.36961 (17)	1.09547 (11)	1.24734 (7)	0.0741 (4)	
H6	1.4319	1.0663	1.2899	0.111*	
C15	1.32138 (13)	0.89438 (10)	1.03809 (7)	0.0316 (2)	
C1	0.67075 (14)	0.51800 (11)	0.93326 (8)	0.0354 (3)	
C2	0.74199 (14)	0.55917 (10)	1.01676 (8)	0.0342 (3)	
H2	0.6960	0.5393	1.0554	0.041*	
C3	0.87346 (13)	0.62569 (10)	1.04157 (7)	0.0299 (2)	
C4	0.94702 (13)	0.65387 (9)	0.98182 (7)	0.0269 (2)	
C5	0.88131 (13)	0.60745 (10)	0.90244 (7)	0.0291 (2)	
C6	0.94786 (14)	0.62367 (11)	0.84276 (7)	0.0341 (3)	
H6A	0.9014	0.5908	0.7903	0.041*	
C7	1.08379 (14)	0.68894 (10)	0.86131 (7)	0.0319 (2)	
C8	1.14982 (13)	0.74657 (9)	0.93879 (7)	0.0286 (2)	
C9	1.07994 (13)	0.72530 (9)	0.99702 (7)	0.0281 (2)	
H9	1.1234	0.7602	1.0489	0.034*	
C10	0.94310 (18)	0.66923 (12)	1.12910 (8)	0.0429 (3)	
H10A	0.8821	0.6405	1.1606	0.064*	
H10B	0.9415	0.7526	1.1291	0.064*	
H10C	1.0503	0.6425	1.1537	0.064*	
C11	1.29393 (13)	0.82800 (10)	0.95799 (7)	0.0318 (2)	
C12	1.26202 (16)	0.92183 (11)	0.88992 (8)	0.0386 (3)	
H12A	1.3578	0.9666	0.8997	0.046*	
H12B	1.2353	0.8842	0.8363	0.046*	
C13	1.13148 (16)	1.00189 (10)	0.88775 (8)	0.0378 (3)	
C14	1.24095 (14)	0.99771 (10)	1.03566 (7)	0.0338 (3)	
C16	1.42053 (15)	0.85861 (11)	1.11672 (8)	0.0393 (3)	
H16	1.4763	0.7894	1.1218	0.047*	
C17	1.43867 (16)	0.92269 (12)	1.18742 (8)	0.0456 (3)	
H17	1.5052	0.8961	1.2390	0.055*	
C18	1.35767 (17)	1.02674 (13)	1.18141 (8)	0.0460 (3)	
C19	1.25766 (17)	1.06437 (12)	1.10452 (8)	0.0428 (3)	
H19	1.2024	1.1338	1.0994	0.051*	
C20	1.44116 (15)	0.75869 (12)	0.96108 (9)	0.0440 (3)	
H20A	1.4596	0.6973	1.0012	0.066*	
H20B	1.5313	0.8094	0.9767	0.066*	
H20C	1.4245	0.7261	0.9073	0.066*	

### Atomic displacement parameters (Å^2^)

**Table:**

	*U*^11^	*U*^22^	*U*^33^	*U*^12^	*U*^13^	*U*^23^
O1	0.0348 (4)	0.0447 (5)	0.0291 (4)	−0.0113 (4)	0.0090 (4)	−0.0025 (4)
O2	0.0374 (5)	0.0640 (6)	0.0427 (5)	−0.0179 (4)	0.0095 (4)	0.0013 (5)
O3	0.0555 (6)	0.0535 (6)	0.0338 (5)	−0.0153 (5)	0.0250 (4)	−0.0071 (4)
O4	0.0494 (5)	0.0416 (5)	0.0294 (4)	0.0115 (4)	0.0082 (4)	0.0055 (4)
O5	0.0673 (7)	0.0513 (6)	0.0321 (5)	0.0083 (5)	0.0099 (5)	0.0129 (4)
O6	0.0944 (10)	0.0718 (8)	0.0366 (6)	0.0264 (7)	−0.0004 (6)	−0.0133 (5)
C15	0.0309 (6)	0.0287 (5)	0.0329 (6)	−0.0062 (4)	0.0087 (5)	0.0019 (4)
C1	0.0316 (6)	0.0374 (6)	0.0360 (6)	−0.0017 (5)	0.0105 (5)	0.0052 (5)
C2	0.0345 (6)	0.0376 (6)	0.0341 (6)	0.0009 (5)	0.0165 (5)	0.0032 (5)
C3	0.0351 (6)	0.0268 (5)	0.0295 (5)	0.0035 (4)	0.0134 (5)	0.0015 (4)
C4	0.0292 (5)	0.0251 (5)	0.0255 (5)	0.0020 (4)	0.0087 (4)	0.0015 (4)
C5	0.0288 (5)	0.0285 (5)	0.0278 (5)	−0.0021 (4)	0.0073 (4)	0.0010 (4)
C6	0.0407 (6)	0.0372 (6)	0.0226 (5)	−0.0054 (5)	0.0091 (5)	−0.0026 (4)
C7	0.0388 (6)	0.0327 (6)	0.0268 (5)	−0.0007 (5)	0.0147 (5)	0.0023 (4)
C8	0.0312 (5)	0.0247 (5)	0.0290 (5)	−0.0001 (4)	0.0099 (5)	0.0021 (4)
C9	0.0329 (6)	0.0254 (5)	0.0246 (5)	0.0005 (4)	0.0085 (4)	0.0002 (4)
C10	0.0587 (8)	0.0417 (7)	0.0325 (6)	−0.0083 (6)	0.0215 (6)	−0.0054 (5)
C11	0.0327 (6)	0.0290 (5)	0.0336 (6)	−0.0028 (4)	0.0116 (5)	0.0023 (4)
C12	0.0486 (7)	0.0342 (6)	0.0373 (6)	−0.0056 (5)	0.0208 (6)	0.0042 (5)
C13	0.0509 (7)	0.0309 (6)	0.0314 (6)	−0.0048 (5)	0.0143 (6)	0.0069 (5)
C14	0.0343 (6)	0.0330 (6)	0.0298 (6)	−0.0015 (5)	0.0063 (5)	0.0050 (5)
C16	0.0359 (6)	0.0341 (6)	0.0402 (7)	0.0004 (5)	0.0040 (5)	0.0033 (5)
C17	0.0428 (7)	0.0480 (7)	0.0334 (6)	0.0006 (6)	−0.0017 (6)	0.0035 (6)
C18	0.0499 (8)	0.0469 (7)	0.0339 (7)	0.0001 (6)	0.0061 (6)	−0.0047 (6)
C19	0.0492 (8)	0.0371 (6)	0.0379 (7)	0.0059 (6)	0.0104 (6)	0.0001 (5)
C20	0.0363 (7)	0.0437 (7)	0.0543 (8)	0.0002 (5)	0.0190 (6)	−0.0004 (6)

### Geometric parameters (Å, °)

**Table:**

O1—C1	1.3646 (14)	C7—C8	1.4176 (16)
O1—C5	1.3751 (13)	C8—C9	1.3844 (15)
O2—C1	1.2166 (15)	C8—C11	1.5424 (15)
O3—C7	1.3604 (13)	C9—H9	0.9300
O3—H3	0.8200	C10—H10A	0.9600
O4—C13	1.3489 (15)	C10—H10B	0.9600
O4—C14	1.4036 (14)	C10—H10C	0.9600
O5—C13	1.2052 (15)	C11—C20	1.5367 (17)
O6—C18	1.3561 (17)	C11—C12	1.5437 (16)
O6—H6	0.8200	C12—C13	1.4876 (19)
C15—C14	1.3871 (17)	C12—H12A	0.9700
C15—C16	1.3936 (17)	C12—H12B	0.9700
C15—C11	1.5180 (16)	C14—C19	1.3749 (18)
C1—C2	1.4308 (17)	C16—C17	1.3824 (19)
C2—C3	1.3492 (16)	C16—H16	0.9300
C2—H2	0.9300	C17—C18	1.388 (2)
C3—C4	1.4472 (15)	C17—H17	0.9300
C3—C10	1.4978 (16)	C18—C19	1.3805 (19)
C4—C5	1.3887 (15)	C19—H19	0.9300
C4—C9	1.4019 (15)	C20—H20A	0.9600
C5—C6	1.3763 (16)	C20—H20B	0.9600
C6—C7	1.3776 (16)	C20—H20C	0.9600
C6—H6A	0.9300		
			
C1—O1—C5	121.03 (9)	H10A—C10—H10C	109.5
C7—O3—H3	109.5	H10B—C10—H10C	109.5
C13—O4—C14	119.83 (10)	C15—C11—C20	111.76 (10)
C18—O6—H6	109.5	C15—C11—C8	110.66 (9)
C14—C15—C16	115.58 (11)	C20—C11—C8	110.33 (10)
C14—C15—C11	119.34 (10)	C15—C11—C12	105.29 (9)
C16—C15—C11	125.07 (11)	C20—C11—C12	108.35 (10)
O2—C1—O1	115.72 (11)	C8—C11—C12	110.32 (10)
O2—C1—C2	126.38 (11)	C13—C12—C11	112.62 (10)
O1—C1—C2	117.90 (10)	C13—C12—H12A	109.1
C3—C2—C1	122.46 (11)	C11—C12—H12A	109.1
C3—C2—H2	118.8	C13—C12—H12B	109.1
C1—C2—H2	118.8	C11—C12—H12B	109.1
C2—C3—C4	118.58 (10)	H12A—C12—H12B	107.8
C2—C3—C10	121.05 (11)	O5—C13—O4	117.23 (12)
C4—C3—C10	120.37 (10)	O5—C13—C12	125.71 (12)
C5—C4—C9	116.57 (10)	O4—C13—C12	117.05 (11)
C5—C4—C3	118.02 (10)	C19—C14—C15	123.86 (11)
C9—C4—C3	125.41 (10)	C19—C14—O4	114.47 (11)
O1—C5—C6	115.82 (10)	C15—C14—O4	121.62 (11)
O1—C5—C4	121.80 (10)	C17—C16—C15	122.11 (12)
C6—C5—C4	122.37 (10)	C17—C16—H16	118.9
C5—C6—C7	119.49 (10)	C15—C16—H16	118.9
C5—C6—H6A	120.3	C16—C17—C18	120.06 (12)
C7—C6—H6A	120.3	C16—C17—H17	120.0
O3—C7—C6	119.98 (10)	C18—C17—H17	120.0
O3—C7—C8	119.00 (10)	O6—C18—C19	116.71 (13)
C6—C7—C8	121.03 (10)	O6—C18—C17	123.94 (13)
C9—C8—C7	116.82 (10)	C19—C18—C17	119.34 (13)
C9—C8—C11	121.40 (10)	C14—C19—C18	119.04 (12)
C7—C8—C11	121.78 (10)	C14—C19—H19	120.5
C8—C9—C4	123.42 (10)	C18—C19—H19	120.5
C8—C9—H9	118.3	C11—C20—H20A	109.5
C4—C9—H9	118.3	C11—C20—H20B	109.5
C3—C10—H10A	109.5	H20A—C20—H20B	109.5
C3—C10—H10B	109.5	C11—C20—H20C	109.5
H10A—C10—H10B	109.5	H20A—C20—H20C	109.5
C3—C10—H10C	109.5	H20B—C20—H20C	109.5
			
C5—O1—C1—O2	175.48 (11)	C16—C15—C11—C8	92.09 (14)
C5—O1—C1—C2	−4.23 (16)	C14—C15—C11—C12	32.87 (14)
O2—C1—C2—C3	−174.86 (13)	C16—C15—C11—C12	−148.70 (12)
O1—C1—C2—C3	4.81 (18)	C9—C8—C11—C15	−10.98 (14)
C1—C2—C3—C4	−1.56 (17)	C7—C8—C11—C15	169.78 (10)
C1—C2—C3—C10	178.78 (12)	C9—C8—C11—C20	113.22 (12)
C2—C3—C4—C5	−2.19 (16)	C7—C8—C11—C20	−66.02 (14)
C10—C3—C4—C5	177.46 (11)	C9—C8—C11—C12	−127.09 (11)
C2—C3—C4—C9	177.44 (11)	C7—C8—C11—C12	53.67 (14)
C10—C3—C4—C9	−2.90 (17)	C15—C11—C12—C13	−54.35 (13)
C1—O1—C5—C6	179.56 (11)	C20—C11—C12—C13	−174.05 (11)
C1—O1—C5—C4	0.53 (16)	C8—C11—C12—C13	65.08 (13)
C9—C4—C5—O1	−176.89 (10)	C14—O4—C13—O5	177.23 (11)
C3—C4—C5—O1	2.77 (16)	C14—O4—C13—C12	−4.36 (16)
C9—C4—C5—C6	4.14 (16)	C11—C12—C13—O5	−138.57 (13)
C3—C4—C5—C6	−176.19 (11)	C11—C12—C13—O4	43.17 (15)
O1—C5—C6—C7	−179.59 (10)	C16—C15—C14—C19	1.57 (18)
C4—C5—C6—C7	−0.57 (18)	C11—C15—C14—C19	−179.86 (12)
C5—C6—C7—O3	175.00 (11)	C16—C15—C14—O4	−175.78 (11)
C5—C6—C7—C8	−4.60 (18)	C11—C15—C14—O4	2.79 (17)
O3—C7—C8—C9	−173.82 (10)	C13—O4—C14—C19	162.46 (12)
C6—C7—C8—C9	5.79 (16)	C13—O4—C14—C15	−19.96 (17)
O3—C7—C8—C11	5.46 (17)	C14—C15—C16—C17	−0.69 (18)
C6—C7—C8—C11	−174.94 (10)	C11—C15—C16—C17	−179.17 (12)
C7—C8—C9—C4	−2.04 (16)	C15—C16—C17—C18	−0.5 (2)
C11—C8—C9—C4	178.69 (10)	C16—C17—C18—O6	−179.39 (15)
C5—C4—C9—C8	−2.76 (16)	C16—C17—C18—C19	0.9 (2)
C3—C4—C9—C8	177.60 (10)	C15—C14—C19—C18	−1.2 (2)
C14—C15—C11—C20	150.29 (11)	O4—C14—C19—C18	176.31 (12)
C16—C15—C11—C20	−31.29 (16)	O6—C18—C19—C14	−179.82 (14)
C14—C15—C11—C8	−86.33 (12)	C17—C18—C19—C14	−0.1 (2)

### Hydrogen-bond geometry (Å, °)

**Table:**

*D*—H···*A*	*D*—H	H···*A*	*D*···*A*	*D*—H···*A*
O3—H3···O5^i^	0.82	2.13	2.9406 (13)	169.
O6—H6···O2^ii^	0.82	1.91	2.7024 (15)	161.

Symmetry codes: (i) −*x*+2, *y*−1/2, −*z*+3/2; (ii) *x*+1, −*y*+3/2, *z*+1/2.
